# Beyond Standard Protocols: Advanced Patented Technology for Comprehensive Toxicity Assessments in Neotropical Bees

**DOI:** 10.3390/toxics14040317

**Published:** 2026-04-09

**Authors:** Adna Suelen Dorigo, Lucas Miotelo, Roberta Cornélio Ferreira Nocelli, Osmar Malaspina, Annelise de Souza Rosa-Fontana

**Affiliations:** 1 Department of General and Applied Biology, Biosciences Institute of Rio Claro, Sao Paulo State University (UNESP), Avenida 24-A, 1515, Rio Claro 13506-900, Brazil; adnadorigoo@gmail.com (A.S.D.); lucas.miotelo@unesp.br (L.M.); osmar.malaspina@unesp.br (O.M.); 2 Center of Agrarian Sciences, Federal University of Sao Carlos (UFSCar), Anhanguera Road Km 174, Araras 13604-900, Brazil; roberta@cca.ufscar.br; 3 Regional Institute for Agri-Food and Forestry Research and Development of Castilla-La Mancha (IRIAF), 19180 Marchamalo, Spain

**Keywords:** in vitro larval rearing method, *Melipona scutellaris*, *Scaptotrigona postica*, thiamethoxam, neonicotinoids, risk assessment, stingless bees

## Abstract

Brazil hosts the world’s greatest stingless bee diversity but remains a leading pesticide consumer. This study evaluated the effects of thiamethoxam on *Melipona scutellaris* (Apidae) and *Scaptotrigona postica* (Apidae) larvae using standardized in vitro protocols and patented biomimetic technologies. Larvae were exposed to a field-realistic dose (RD) of 0.02292 ng a.i./larva—calculated using the BeeRex model for citrus crops—and two lower doses: RD/10 and RD/100. Thiamethoxam exposure resulted in significant mortality and developmental alterations, even at 100-fold dilutions. In *M. scutellaris*, mortality was dose-dependent; RD and RD/10 induced body malformation and reduced food consumption, resulting in >98% mortality. At RD/100, surviving individuals showed significant reductions in body size. In *S. postica*, all tested doses induced larval darkening and accelerated fungal growth, leading to 100% mortality during the feeding period, including at RD/100. This pattern contrasts with the greater tolerance reported for the adult stage of this species. Overall, the results suggest that larval stages may be more sensitive to thiamethoxam exposure than adults, highlighting the importance of considering different life stages in pesticide risk assessment. These findings also emphasize the need for validated experimental approaches to support environmental risk evaluation for Neotropical pollinators.

## 1. Introduction

Stingless bees (Hymenoptera: Apidae: Meliponini) are essential pollinators in Neotropical ecosystems, playing a fundamental role in maintaining biodiversity and ensuring the productivity of numerous agricultural crops [1]. Brazil possesses the greatest diversity of native stingless bees in the world; however, the country’s status as a global leader in pesticide use poses a constant threat to these pollinators [2,3].

In response to this threat, efforts to investigate the suitability of *Apis mellifera* Linnaeus, 1758 as a universal surrogate for wild bees have intensified [4,5,6]. Research has highlighted the importance of including non-*Apis* species, particularly stingless bees, in environmental risk assessments (ERAs) [7,8,9]. To address this gap in Brazil, collaborative efforts between the Laboratory of Ecotoxicology and Bee Conservation (UNESP) and Bees and Environmental Services (UFSCar) have focused on adapting and standardizing toxicological methods for native species [10,11,12].

Current ERA frameworks are predominantly centered on *A. mellifera* as the universal surrogate. However, evidence suggests that this model may underestimate risks, as several wild bee species have been identified as significantly more sensitive to pesticides than honeybees in the adult stage [5,13,14]. Furthermore, even within the Meliponini tribe, studies have demonstrated significant interspecific variation in sensitivity. Notably, Lourencetti et al. (2023) reported that *Melipona scutellaris* Latreille, 1811 exhibits higher susceptibility to the commercial formulation of thiamethoxam compared to *Scaptotrigona postica* Latreille, 1807 in the adult phase, with the latter often being identified as a more tolerant species in comparative assessments [15]. For the active ingredient thiamethoxam, reported lethal concentration (LC_50_) values increase from *Scaptotrigona bipunctata* (0.002 ng/µL) (Apidae), *M. scutellaris* (0.0543 ng/µL), *S. postica* (0.11 ng/µL), and *A. mellifera* (0.227 ng/µL) to *Friesiomelitta varia* (0.68 ng/µL) (Apidae), indicating that several stingless bee species show higher sensitivity to this insecticide than *A. mellifera* [16,17,18,19].

Nevertheless, relying solely on adult data can be misleading; the application of the *Apis* model is particularly inappropriate for the larval phase due to the contrasting larval ontogeny and feeding systems [10,11]. While *A. mellifera* larvae are fed progressively, stingless bees rely on mass provisioning, where the entire larval food supply is deposited at once before oviposition [20]. Consequently, stingless bee larvae are particularly vulnerable as they are confined with a fixed amount of potentially contaminated food for their entire development.

According to the guidelines of the Organisation for Economic Co-operation and Development (OECD), which establish internationally standardized protocols for chemical toxicity testing and environmental risk assessment, toxicological comparisons among species require standardized experimental methods; however, such comparisons are often unfeasible in this context. While honeybee protocols consider either single [21] or continuous exposure [22], stingless bees undergo a unique dual-exposure process: a single initial offer of the active ingredient followed by continuous consumption over consecutive days [10,11]. This biological divergence underscores the potential limitations in using *Apis* as a surrogate and emphasizes the need for specialized, species-specific testing frameworks, especially for neonicotinoids like thiamethoxam. Thiamethoxam is a systemic neonicotinoid insecticide widely used in agricultural crops such as citrus, soybean, and maize [23]. Like other neonicotinoids, it acts as an agonist of nicotinic acetylcholine receptors in the insect nervous system, causing continuous neural stimulation that can lead to paralysis and death. As a systemic compound, thiamethoxam can translocate to nectar and pollen, leading to chronic colony exposure [24]. While lethal effects are critical, such sublethal exposure can trigger developmental delays and permanent morphological impairments that compromise colony fitness [25,26].

The baseline sensitivity of *S. postica* to thiamethoxam was initially established through toxicity protocols using conventional ELISA plates [26], paving the way for further in-depth assessments [25]. However, these traditional methods utilize standard plasticware that fails to provide the precise dimensions and microenvironmental conditions of natural brood cells. Such spatial constraints can induce experimental artifacts and limit the accurate monitoring of larval development and sublethal physiological responses.

To address these methodological limitations, the present study employed patented custom-designed acrylic plates [27], engineered to approximate the dimensions of natural brood cells of *M. scutellaris* and *S. postica*. This biomimetic system allows standardized evaluation of pesticide toxicity in stingless bees during the larval phase. Using this approach, we assessed the effects of thiamethoxam by analyzing survival, developmental time, and morphometric parameters under exposure to the recommended field dose (RD) and its dilutions (RD/10 and RD/100). The results contribute to the understanding of pesticide effects on Neotropical stingless bees and provide data relevant for environmental risk assessment.

## 2. Materials and Methods

### 2.1. Bee Provenance and In Vitro Rearing

Three non-parental colonies of *M. scutellaris* and *S. postica* were used. The colonies were maintained at the meliponary (geographic coordinates 22°23′49″ S and 47°32′37″ W) of the São Paulo State University (UNESP), Brazil. Larvae were obtained directly from brood combs of these colonies and were not derived from laboratory-reared generations. Larval rearing followed the protocols described by Dorigo et al. (2019) for *M. scutellaris* [10] and by Rosa-Fontana et al. (2020) for *S. postica* [11].

**Figure 1:**
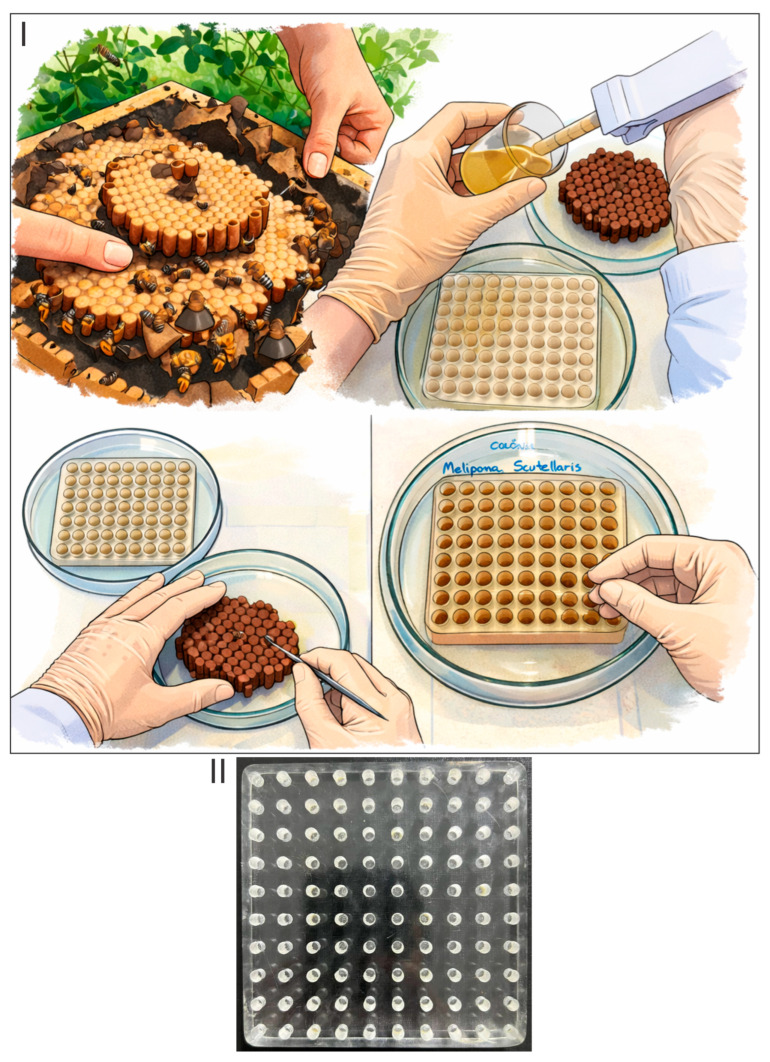
Workflow of brood comb collection and the in vitro larval rearing procedure for stingless bees. (**I**) Colonies maintained in wooden boxes at the meliponary of São Paulo State University (UNESP) were used as the source of brood combs. Combs were collected for two purposes: obtaining larval food and collecting first-instar larvae. In the laboratory, pesticide-free larval food was separated for the control group, while larval food used for the exposed groups received the addition of thiamethoxam to reach the final concentrations of 0.02292 (RD), 0.002292 (RD/10), and 0.0002292 (RD/100). First-instar larvae were carefully transferred to acrylic plates containing larval food. The plates were placed inside glass Petri dishes and maintained in a biochemical oxygen demand (BOD) incubator at 30 °C and 95% relative humidity. Larval mortality was recorded daily until adult emergence. (**II**) Acrylic plate used in the assays. The specific dimensions and the detailed methodological description are available in the protocols proposed by Dorigo et al. (2019) for *Melipona scutellaris* and Rosa-Fontana et al. (2020) for *Scaptotrigona postica* [10,11].

While standard in vitro protocols typically utilize commercial ELISA plates, which are primarily optimized for the larger larvae of *A. mellifera*, this study employed patented custom-designed acrylic plates [27]. These plates were designed to better approximate the natural architecture of stingless bee brood cells. The workflow of brood comb collection and the in vitro larval rearing procedure for stingless bees are shown in Figure 1. The dimensions (height and diameter) of the plate cavities were determined according to species-specific morphometric measurements of natural brood cells established in previous studies [10,11].

Newly hatched larvae were carefully transferred to these customized biomimetic plates and kept in an incubator under controlled conditions (28 ± 1 °C and 95% relative humidity). The experiments were conducted in triplicate. For *M. scutellaris*, 30 larvae were used per treatment group (control, RD, RD/10, and RD/100). For *S. postica*, 60 larvae were used per treatment group, following species-specific protocols described in previous studies [10,11].

### 2.2. Dietary Pesticide Exposure

The active ingredient (a.i.) thiamethoxam (C_8_H_10_ClN_5_O_3_S) was obtained in analytical standard (Pestanal^®^, Sigma-Aldrich, Saint Louis, MO, USA) with 98% purity and high water solubility. For both species, a stock solution of 1000 ng a.i./µL was prepared in deionized water, and serial dilutions were made in the larval food to obtain the following doses offered to the larvae (ng active ingredient per larvae): 0.02292 (RD), 0.002292 (RD/10), and 0.0002292 (RD/100). The doses were calculated from the field-recommended dose (RD) for citrus application according to the Brazilian Ministry of Agriculture and Livestock. Residual values in pollen and nectar were estimated using the BeeRex tool (Version 1.0; 30 October 2015) [28].

### 2.3. Performance Assessment and Morphometry

For *M. scutellaris*, we assessed emergence and mortality rates, developmental time, and the morphometry of newly emerged workers. For *S. postica*, morphometric analysis was not performed, as no individuals from the treated groups reached the adult stage. Morphometric parameters included intertegular distance and head width, measured using ImageJ software (Version 1.54g).

### 2.4. Statistical Analysis

Data are expressed as mean ± standard error (SE) for the following parameters: (1) emergence rate relative to transferred larvae, (2) emergence rate relative to the number of pupae, and (3) larval mortality. Data normality was verified using the Shapiro–Wilk test, and homogeneity of variances was assessed using Bartlett’s test. Groups were compared using One-Way ANOVA followed by Tukey’s post hoc test for multiple comparisons. For non-parametric data, the Kruskal–Wallis test was applied. Morphometric means (intertegular distance and head width) were compared using Student’s *t*-test for independent samples.

Survival analyses were performed using the Kaplan–Meier estimator, and differences among treatments were assessed using the Log-rank test. Furthermore, treatment effects on mortality risk were evaluated using Cox proportional hazards models. To account for potential non-independence among individuals originating from the same experimental plate, the plate was included as a clustering factor, and robust standard errors were calculated. Hazard ratios (HRs) and 95% confidence intervals (CIs) were reported to express the instantaneous mortality risk. All analyses were conducted in R software (version 4.3.2) using the survival and survminer packages, with a significance level set at 0.05.

## 3. Results

### 3.1. Developmental Success and Survival of M. scutellaris

#### 3.1.1. Developmental Rates

Thiamethoxam exposure significantly impaired the developmental success of *M. scutellaris* (Table 1). High-concentration treatments (RD and RD/10) resulted in a substantial increase in larval mortality, reaching 90% in both groups, a value significantly higher than that observed in the Control and RD/100 groups (One-way ANOVA, *p* < 0.001). This high larval mortality was accompanied by a marked reduction in pupal success rate and adult emergence, which decreased to approximately 10–16% with the higher doses (Tukey’s test, *p* < 0.05). While the lowest concentration (RD/100) did not differ statistically from the control in these overall rates, additional effects were detected in the survival and morphometric analyses.

#### 3.1.2. Survival Dynamics and Mortality Risk

**Figure 2:**
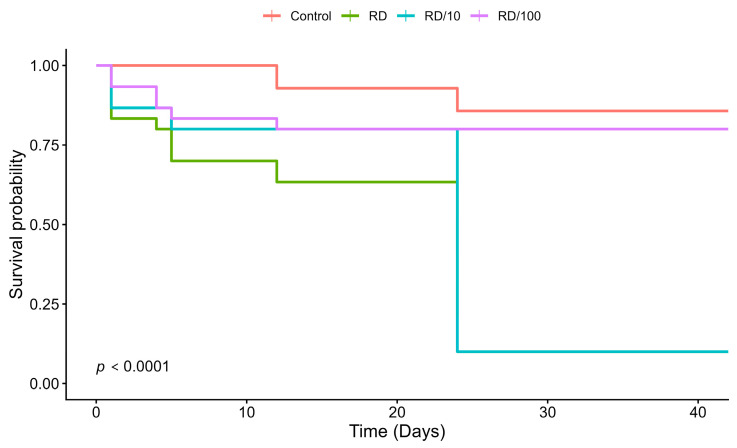
Survival probability of *M. scutellaris* larvae exposed to thiamethoxam. Kaplan–Meier survival curves showing larval survival over 44 days under exposure to field-realistic doses of thiamethoxam. Different colors represent the treatments: Control (red), RD/100 (purple), RD/10 (blue), and RD (green). The “RD” (Recommended Dose) corresponds to the field-realistic concentration, with RD/10 and RD/100 representing 10-fold and 100-fold dilutions, respectively. Survival curves were compared using the Log-rank (Mantel–Cox) test. Each treatment group consisted of 30 larvae.

Beyond the final developmental rates, survival probability over time was significantly affected by thiamethoxam exposure (Log-rank test: χ^2^ = 49.2, df = 3, *p* < 0.0001; Figure 2). The control group exhibited high viability (mortality < 15%), and all individuals appeared morphologically normal. In contrast, a dose-dependent pattern was observed; bees exposed to RD and RD/10 were 13.9 and 12.1 times more likely to die than control individuals, respectively (Cox model, *p* < 0.001).

In these high-exposure groups, larvae exhibited body darkening (melanization) and reduced food consumption as early as the 4th day of development, leading to a median survival of 24 days. The Cox proportional hazards model also indicated a significant increase in mortality risk at the lowest concentration (RD/100; HR = 1.81; 95% CI = 1.07–3.03; *p* = 0.025), an effect not detected in the developmental percentages shown in Table 1.

#### 3.1.3. Morphometric Sublethal Effects

Due to the high mortality rates in the RD and RD/10 groups, only individuals from the RD/100 treatment emerged in sufficient numbers to allow morphometric comparison with the control group. As previously noted, individuals exposed to higher doses failed to complete metamorphosis, preventing their inclusion in this analysis.

**Figure 3:**
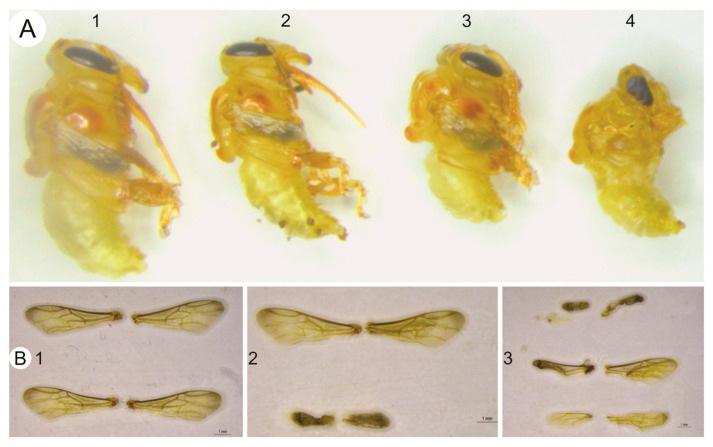
Sublethal effects of thiamethoxam on the morphology of *M. scutellaris* workers. (**A**) Comparative body size of workers at the 37th day of development (1: Control, 2: RD/100, 3: RD/10, and 4: RD). (**B**) Forewings of workers at the 37th day of development. From top to bottom (1: two pairs from the control group; 2: comparison between control and RD; and 3: comparison between RD, RD/10, and RD/100, illustrating the progressive reduction in wing development and area).

A reduction in body size was observed by the 37th day of development in individuals exposed to thiamethoxam (Figure 3A). Quantitative analysis showed that exposure to the lowest dose (RD/100) resulted in smaller body parameters compared with the control group. The mean intertegular distance decreased from 2.39 mm in the control group to 2.21 mm in treated bees. Similarly, mean head width decreased from 3.31 mm in control individuals to 3.14 mm in those exposed to the insecticide.

Student’s *t*-test confirmed that these differences were significant for both parameters (*p* < 0.001). In addition, alterations in forewing development and size were observed in the treated group compared with the control (Figure 3B), indicating that individuals reaching adulthood after low-dose exposure exhibited morphological differences relative to control bees.

### 3.2. Development and Survival of S. postica

**Figure 4:**
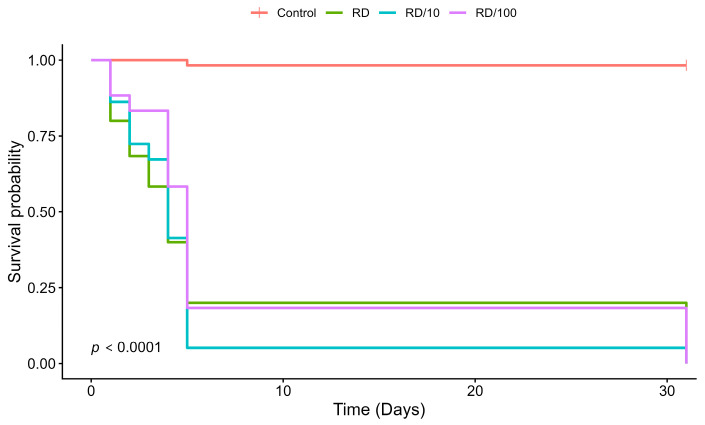
Survival probability of *S. postica* larvae exposed to thiamethoxam. Kaplan–Meier survival curves showing larval survival over 31 days under exposure to field-realistic doses of thiamethoxam. Different colors represent the treatments: Control (red), RD/100 (purple), RD/10 (blue), and RD (green). The “RD” (Recommended Dose) corresponds to the field-realistic concentration, with RD/10 and RD/100 representing 10-fold and 100-fold dilutions, respectively. Survival curves were compared using the Log-rank (Mantel–Cox) test. Each treatment group consisted of 60 larvae.

The survival of *S. postica* was significantly affected by thiamethoxam exposure (Log-rank test: χ^2^ = 166, df = 3, *p* < 0.0001; Figure 4). Compared with the control group, which showed high survival and successful development (mortality < 2%), all insecticide-treated groups showed a marked reduction in survival. The Cox proportional hazards analysis, accounting for the experimental plate as a clustering factor, indicated a substantial increase in mortality risk: larvae exposed to RD, RD/10, and RD/100 presented instantaneous mortality risks approximately 322, 408, and 273 times higher than the control group, respectively (Wald test, *p* < 0.001).

**Figure 5:**
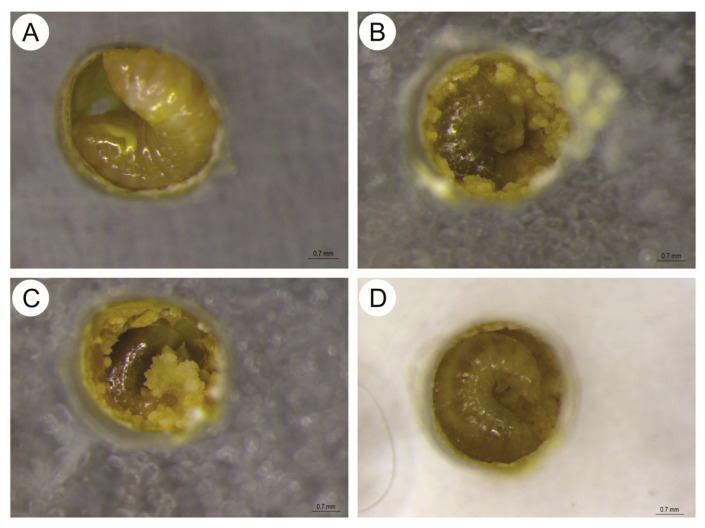
Developmental changes and mortality in S. postica larvae under thiamethoxam exposure. (**A**) Control group showing healthy larval development and normal consumption of the diet. (**B**) RD treatment, (**C**) RD/10 treatment, and (**D**) RD/100 treatment. Note the characteristic body melanization (darkening), feeding inhibition with residual food, and fungal proliferation within the brood cells in all thiamethoxam-treated groups.

This pattern was also reflected in the median survival times, which were reduced to 4 days for the RD and RD/10 groups, and 5 days for the RD/100 group. Unlike *M. scutellaris*, all tested concentrations resulted in mortality in *S. postica*, with deaths occurring as early as the 2nd and 3rd days of exposure. Treated larvae exhibited progressive body darkening (melanization) and reduced or absent food consumption (Figure 5B–D). Fungal growth was also observed in the brood cells of treated individuals.

By the end of the feeding period (120 h), more than 80% of the larvae in all thiamethoxam treatments had died. By the 31st day, nearly all control bees had successfully emerged, whereas complete mortality (100%) was recorded in all treated groups. Pairwise comparisons confirmed that the lowest concentration (RD/100) also resulted in complete mortality (*p* < 0.001), with no significant differences detected among the three insecticide doses (*p* > 0.05). Because all treated individuals died during the larval stage, it was not possible to calculate pupation or emergence rates.

## 4. Discussion

### 4.1. Larval and Adult Sensitivity Differences Between Species

Our findings indicate a difference in sensitivity between larval and adult stages of *S. postica*. Previous studies on adult stingless bees exposed to thiamethoxam identified *S. postica* as a relatively tolerant species compared with *M. scutellaris* [15]. However, the present results suggest that this tolerance may be stage-dependent and may not extend to the larval phase. In the present study, *S. postica* larvae showed high sensitivity, with 100% mortality observed even at the lowest concentration tested (RD/100).

Previous studies with the active ingredient thiamethoxam reported a survival time of 5 days for *A. mellifera* forager bees exposed to a concentration equivalent to 10-fold the LC_50_ (0.0227 ng/µL) [18]. Among stingless bees, *F. varia* showed an LT_50_ of 25 days (LC_50_/10, 0.068 ng/µL) and 27 days (LC_50_/100, 0.0068 ng/µL) [20], *S. postica* 8 days (LC_50_/10, 0.011 ng/µL) and 15 days (LC_50_/100, 0.0011 ng/µL) [18], and *M. scutellaris* 3 days (LC_50_/10, 0.00543 ng/µL) and 7 days (LC_50_/100, 0.000543 ng/µL) [29]. These results indicate that exposure to thiamethoxam can lead to substantial mortality in stingless bees, even at fractions of the LC_50_ commonly used to assess sublethal effects, suggesting that this insecticide may represent a potential risk to non-target pollinators.

### 4.2. Standardization and Reproducibility via Biomimetic Technology

This study employed patented custom-designed acrylic plates [27], which allowed controlled in vitro rearing conditions for stingless bee larvae. Traditional in vitro protocols often utilize standard ELISA plates which, although widely available, may not fully reproduce the spatial and microenvironmental conditions required for stingless bee larval development. Using a biomimetic approach, the dimensions and architecture of natural brood cells of *M. scutellaris* and *S. postica* were approximated according to previously established morphometric measurements [10,11].

This approach helped ensure that the observed mortality and developmental delays were associated with thiamethoxam exposure rather than potential artifacts related to spatial constraints or gas exchange limitations. In addition, the transparency of the acrylic plates allowed daily monitoring of larval development. For example, body melanization and reduced food consumption in *S. postica* larvae could be detected as early as the fourth day of development.

The standardization provided by this biomimetic framework may also contribute to improving the reproducibility of toxicity assays for Neotropical stingless bees. By reducing variability during larval development, this system provides a framework for evaluating pesticide toxicity in stingless bee larvae. Such methodological consistency is important for the implementation of interlaboratory ring tests, which are necessary for the validation of new toxicological protocols [30]. These advances may support future efforts to include stingless bee larvae in environmental risk assessment frameworks.

### 4.3. Morphological Anomalies and Behavioral Alterations

In addition to mortality, the biomimetic plates allowed the observation of phenotypic indicators associated with thiamethoxam exposure. The most immediate behavioral alteration observed was feeding inhibition, characterized by a significant amount of residual food in the cells of treated larvae. This finding aligns with the histological damage previously reported for *S. postica* [17], where neonicotinoid exposure led to the progressive degeneration of the midgut (ventriculus) epithelium. If the digestive tract is structurally compromised, the larvae lose the ability to assimilate nutrients, which may contribute to larval mortality despite the presence of food.

Furthermore, such pesticide-induced nutritional stress has been shown to trigger a “caste-shift” in Neotropical species like *Plebeia droryana* [31], where surviving larvae destined to become queens developed into workers because they consumed less food than that required for queen differentiation. This type of interference in caste determination suggests that the feeding inhibition observed in our trials could not only produce smaller workers but also jeopardize the production of new queens, potentially affecting colony reproduction and its ability to form new nests.

Specifically in *S. postica*, the cessation of feeding was followed by intense body melanization. Potentially, insecticides could interfere with the melanization process either by disrupting serine protease activity or by interfering with the prophenoloxidase cascade. This is a systemic immune response to severe physiological stress, pathogens, or cellular damage [32]. In this context, the widespread darkening of the larval body serves as a visible morphophysiological indicator of physiological stress associated with thiamethoxam exposure. Furthermore, the high humidity required for larval development, combined with the presence of residual food and weakened larval immunity, likely triggered a disruption in the larval–microbe interaction. While *Zygosaccharomyces* fungi are often essential symbionts in the brood cells of *Scaptotrigona* genus [33], providing critical nutritional support, these adverse conditions can cause them to proliferate uncontrollably or act as opportunistic pathogens. This fungal overgrowth, observed uniquely in the *S. postica* bioassays, may have contributed to the mortality observed in the larvae. This immune vulnerability is supported by molecular evidence in other stingless bees, such as *F. varia*, where sublethal pesticide exposure significantly downregulated the expression of antimicrobial peptide genes like *abaecin*, compromising the larva’s ability to maintain homeostasis [34]. The disruption of these microbial associations is a recurring theme in neonicotinoid toxicology across diverse insect taxa. Pesticide-induced immunosuppression often facilitates the transition of beneficial or commensal microbes into opportunistic pathogens, a phenomenon also recorded in various agricultural pests and beneficial insects, where chemical stress shifts the microbiome composition and compromises the host’s primary defenses [35].

Another relevant morphological effect was the occurrence of developmental anomalies in *M. scutellaris* survivors. The observation of individuals on the 37th day exhibiting a “hybrid” state—with the anterior region in the pupal stage while the posterior remained as a larva—suggests a possible disruption in the endocrine signaling that regulates metamorphosis. These incomplete metamorphoses indicate that thiamethoxam interferes with the precise synchronization of ecdysteroids and juvenile hormone levels required for successful molting [36]. Such disruptions in the synchronization of ecdysteroids and juvenile hormone levels are not unique to bees; similar developmental impairments and ecdysis failures have been documented in other insect orders exposed to neonicotinoids, such as Lepidoptera and Coleoptera, where thiamethoxam and related compounds interfere with larval-to-pupa transition and adult emergence [37,38]. Such permanent impairments, including wing atrophy and reduced body size (intertegular distance and head width), suggest that even the individuals that reached adulthood with field-realistic doses carry significant functional deficits.

Similar morphometric reductions and developmental instabilities have been reported across the Meliponini tribe. In *S. bipunctata*, larval exposure to organophosphates resulted in lighter and smaller workers [36], while in *Melipona quadrifasciata anthidioides*, pesticide exposure has been linked to increased fluctuating asymmetry [39], a clear indicator of morphogenetic disruption during development. Furthermore, sublethal doses of biopesticides in *M. quadrifasciata* adversely affected pupal body mass and induced severe malformations that compromised adult walking activity [40].

Such morphological alterations may also be associated with functional impairments later in life. In *A. mellifera*, larval exposure to pesticides has been linked to behavioral alterations in adults, including reduced learning ability [41]. Although behavioral assays were not conducted in the present study, the morphological alterations observed here may indicate potential long-term consequences for worker performance and colony dynamics.

### 4.4. Implications for ERA

The high sensitivity observed in this study, particularly for *S. postica*, suggests that current ERA frameworks may not fully capture the vulnerability of Neotropical stingless bees. Previous studies using field-realistic doses of imidacloprid in *M. quadrifasciata* [42] and thiamethoxam in *S. depilis* [26] have already reported substantial developmental impairments. In the present study, *S. postica* exhibited complete mortality during development even at RD/100. These results suggest that thresholds estimated by models such as BeeRex, which are largely derived from *A. mellifera* parameters, may not adequately represent the sensitivity of stingless bee larvae.

Moreover, the extreme sensitivity of S. postica larvae parallels findings in other non-target taxa, including aquatic invertebrates and predatory insects, where thiamethoxam concentrations previously considered negligible have been shown to cause significant population declines and developmental instability, further questioning the broader applicability of current regulatory thresholds [43].

Furthermore, the occurrence of severe developmental anomalies, where individuals on the 37th day exhibited an intermediate state, with the anterior region in the pupal stage while the posterior remained larval, suggests disruption in the physiological regulation of metamorphosis [36]. Similar developmental alterations have been reported in other bee species [41,44,45], reinforcing the limitations of relying solely on adult-stage toxicity data to predict colony-level effects. These findings highlight the importance of incorporating larval endpoints when evaluating pesticide risks to stingless bees, particularly in Neotropical ecosystems where meliponine species represent key pollinators.

## 5. Conclusions

Our findings demonstrate that the larval stage of Neotropical stingless bees represents a highly sensitive developmental stage under pesticide exposure. By employing patented biomimetic acrylic plates, we established a standardized platform that revealed a stage-dependent pattern of toxicity: *S. postica*, previously considered relatively tolerant as an adult, exhibited high susceptibility during the larval phase, reaching 100% mortality at field-realistic concentrations (RD/100). The observed feeding inhibition, followed by body melanization and fungal proliferation, may represent observable indicators of severe physiological stress in this species, whereas *M. scutellaris* individuals that completed development presented abnormalities such as incomplete metamorphosis and morphological alterations.

Our results indicate that larval exposure to thiamethoxam can lead to adults presenting morphological impairments, including wing atrophy and reductions in morphometric parameters. Such alterations may affect worker performance at the colony level. These findings also suggest that ERA approaches largely derived from adult honeybee data may underestimate potential risks to native stingless bees. The use of standardized biomimetic rearing systems may therefore contribute to improving future regulatory assessments by facilitating the inclusion of larval endpoints in pesticide risk evaluation for Neotropical pollinators.

## 6. Patents

Granted patent: Rosa-Fontana, A.S.; Dorigo, A.S. (Inventors); co-inventors: Nocelli, R.C.F.; Malaspina, O. Register: BR1020220011354. Acrylic plates for in vitro rearing of stingless bees. National Institute of Industrial Property, Brazil. 2022; owner: UNESP and authors. The patented technology is currently exploited by Contract Research Organizations (CROs) and institutions/universities.

## Figures and Tables

**Table 1:** Emergence and mortality rates (Mean ± SE) of *M. scutellaris* larvae orally exposed to thiamethoxam.

Treatment	Larval Mortality (%) *	Pupa Success Rate (%) *	Adult Emergence (%) *
Control	14.44 ± 3.90 a	85.56 ± 3.90 a	100.00 ± 0.00 a
RD/100	23.33 ± 6.67 a	76.67 ± 6.67 a	95.83 ± 4.17 a
RD/10	90.00 ± 5.77 b	10.00 ± 5.77 b	10.83 ± 5.83 b
RD	90.00 ± 0.00 b	10.00 ± 0.00 b	15.88 ± 0.79 b

* Data are expressed as Mean ± Standard Error (SE). Different letters within the same column indicate significant differences among treatments (One-way ANOVA followed by Tukey’s post hoc test). The overall ANOVA results were: Larval mortality (F = 73.07, *p* < 0.001), Pupa success rate (F = 73.07, *p* < 0.001), and Adult emergence (F = 183.83, *p* < 0.001).

## Data Availability

All data associated with this research publication are available from Adna Suelen Dorigo (adnadorigoo@gmail.com) and from Annelise de Souza Rosa-Fontana (annesouzar@gmail.com).
